# Type-I interferon secretion in the acute phase promotes *Cryptococcus neoformans* infection-induced Th17 cell polarization *in vitro*

**DOI:** 10.3892/etm.2014.1517

**Published:** 2014-01-30

**Authors:** HAI-JUN QIN, QI-MING FENG, YONG FANG, LEI SHEN

**Affiliations:** 1 Department of Emergency, Shanghai Jiao Tong University Affiliated Sixth People’s Hospital, Shanghai 200233, P.R. China; 2 Department of Critical Care Medicine, Huashan Hospital, Fudan University, Shanghai 200040, P.R. China

**Keywords:** cryptococcus infection, type-I interferon, interleukin-17A

## Abstract

Cryptococcosis is a potentially fatal fungal disease commonly identified in patients with acquired immunodeficiency syndrome. Cryptococcus infection induces strong pro-inflammatory cytokine secretion, i.e. type-I interferon (IFN-I) via the Toll-like receptor signaling pathway. However, innate immune responses are insufficient in host defense against fungi infection and the clearance of Cryptococcus is dependent on the T helper (Th)17 cell-mediated mucosal immune response. In this study, IFN-I was identified as the early response cytokine to *Cryptococcus neoformans* infection via quantitative PCR (qPCR) and IFN-I was demonstrated to be crucial for interleukin (IL)-17A secretion in T cells, but not in innate immune cells. In addition, blockade of IFN-I reduced the protein expression levels of IL-22 and IL-23 in Th17 cells *in vitro*. These results suggest additional functions of IFN-I in immune regulation, which may be pivotal for the development of clinical immune therapy.

## Introduction

Cryptococcosis, or cryptococcal disease, is a potentially fatal fungal disease caused by *Cryptococcus neoformans* or *Cryptococcus gattii* in patients with acquired immunodeficiency syndrome. Cryptococcal meningoencephalitis results in >600,000 fatalities every year (1). As a fungal infection, the limitation and clearance of cryptococcosis is mostly dependent on innate immune cells, such as dendritic cells (DC), natural killer cells (NK) and neutrophils. Briefly, Cryptococcus infection induces secretion of pro-inflammatory cytokines, including type-I interferon (IFN-I), tumor necrosis factor-α, interleukin (IL)-1β and IL-6, from innate immune cells (2). Studies have suggested that myeloid differentiation primary response gene 88 (MyD88)-mediated Toll-like receptor (TLR) signaling pathways, such as TLR2, TLR4 and TLR9, are involved in this secretory process (3,4). However, in a more recent study, TLR4 was observed to not be required for host defense against cryptococcal infection (5). In addition, Ley *et al* (6)suggested that TLR signals have a limited role in the clearance of Cryptococcus, as MyD88- and TLR-deficient mice were able to survive following cryptococcal infection (6).

By contrast, the roles of adaptive immune cells in the generation of protective anti-cryptococcal infection immune responses have been widely accepted. Studies have shown that increased IL-17A production, a pro-inflammatory cytokine which is predominantly secreted by CD4^+^ T cells, is associated with cryptococcal burden (5,7). Furthermore, IL-17A secreted by CD4^+^ T helper (Th)17 cells is involved in multiple roles as a ‘bridge’ that is associated with innate and adaptive immune responses. Its primary functions include inducing pro-inflammatory secretion in mucosal tissues (8) as a potent inducer. IL-17A can also enhance neutrophil chemotaxis by upregulating the production of granulocyte-colony stimulating factor and chemokine (C-X-C motif) ligand 1 (9). An increased Th17 cell population has also been found to inhibit the generation of Treg cells (10) and promote the clearance of fungal infections. Notably, IL-17A is also secreted by other cells, such as γδ T cells, CD8^+^ T cells, NK T cells, NK cells and neutrophils (11–13).

Differentiation of Th17 cells is dependent on IL-6 and transforming growth factor-β (TGF-β) signals; however, MyD88 was previously identified to enhance Th17 polarization by inducing IL-1β and IL-23 production (14). Activation of TLR signals in antigen-presenting cells (APCs) skewed Th subset development in the thymus and the peripheral tissues (15). Additionally, endotoxin exposure, which can specifically activate TLR4 signaling pathways in the *Blomia tropicalis* allergens infection model, shifted Th2 cell response towards the Th17 cell-mediated immune response (16). These results indicate that TLR signaling may be involved in Th17 cell-mediated host anti-cryptococcal infection, particularly in Th17 cell differentiation. In the present study, the effect of *C. neoformans-induced release* of IFN-I on immune regulation was determined. TLR signaling pathways were blocked and IFN-I secretion was examined with neutralizing antibodies.

## Materials and methods

### Cell culture and Pam3CSK4 treatment

Human peripheral blood mononuclear cells (PBMCs) were cultured in Dulbecco’s modified Eagle’s medium (Gibco-BRL, Carlsbad, CA, USA) supplemented with 10% heat-inactivated fetal calf serum, 100 U/ml penicillin and 100 μg/ml streptomycin, then incubated at 37°C in 5% CO_2_. Cultured cells were treated with 0.1 μg/ml tripalmitoyl-*S*-glyceryl-cystein (Pam3CSK4; 910.5 Da; EMC microcollections; Tübingen, Germany) in the medium to activate the TLR2 signaling pathway.

### Cryptococcus strains and culture conditions

The encapsulated *C. neoformans* strain 11959 (ATCC 90112) was cultured in medium containing 1% yeast extract, 1% peptone and 2% glucose at 30°C for 2 days. The cell suspension was mixed with glycerine and stored at −80°C. *C. neoformans* was heat-killed at 65°C for 30 min and 10^9^ fungi/ml medium was added to the co-culture.

### Quantitative polymerase chain reaction (qPCR)

Total RNA was harvested from cells, treated with DNase and reverse transcribed as previously described (17). Universal human (h)IFN-I primers were as follows: Forward, 5′-ATG GCT AGR CTC GTG CTT TCC T-3′ (R is a wobble of A or G) and reverse, 5′-AGG GCT CTC CAG AYT TCT GCT CTG-3′ (Y is a wobble of C or T); hIFN-γ forward, 5′-TCA AGT GGC ATA GAT GTG GAA-3′ and reverse, 5′-CAC TCG GAT GAG CTC ATT GA-3′; hIL-1β forward, 5′-AAA CCT CTT CGA GGC ACA AG-3′ and reverse, 5′-CTG TTT AGG GCC ATC AGC TT-3′; hIL-6 forward, 5′-GAC AAC TTT GGC ATT GTG G-3′ and reverse, 5′-ATG CAG GGA TGA TGT TCT G-3′; hIL-17A forward, 5′-CTG TGT CTC TGA TGC TGT TG-3′ and reverse, 5′-ATG TGG TGG TCC AGC TTTC-3′; and hGAPDH*,* forward 5′-ACC ACA GTC CAT GCC ATC AC-3′ and reverse, 5′-CAC CAC CCT GTT GCT GTA GCC-3′. Relative abundance of each cDNA was normalized to corresponding GAPDH levels and quantified using the ΔCT method.

### Cytokine assays

T-cell derived cytokine levels, IL-17A, IL-22 and IL-23, were determined in the cultured supernatant after 7 days of incubation using commercially available ELISA kits (R&D Systems, Minneapolis, MN, USA) according to the manufacturer’s instructions. Lower detection limits were 40 and 78 pg/ml for IL-17 and IL-22/23, respectively.

### Blockade of IFN-I signal with antibody in mice splenocytes

Splenocytes were isolated from the spleen of female C56Bl/6 mice and treated with isotype antibody or anti-IFN-IR1 antibody 0.1 mg/ml (Invitrogen, Carlsbad, CA, USA).

### Statistical analysis

Results from at least three repeat experiments were pooled and analyzed using GraphPad Prism 5 software (GraphPad Software, Inc., San Diego, CA, USA). Data are expressed as the mean ± standard error. P<0.05 was considered to indicate a statistically significant difference.

## Results

### Cryptococcal infection induces early IFN-I expression prior to Th17 cell activation in vitro

*C. neoformans* infection was established *in vivo* with co-cultured human PBMCs and heat-killed *C. neoformans in vitro*. As previously reported, Cryptococcus infection induces pro-inflammatory cytokine secretion in innate cells and is mostly independent on peroxisome proliferator-activated receptor (PPAR), and other factors including TLR family, MyD88 and NF-κB were reported activated in the clearance of *C. neoformans*. In the present study, the production of TLR signaling molecules and the levels of IFN-I and IFN-γ were examined following *C. neoformans* infection, as well as various cytokines that are critical for Th17 cell differentiation, such as IL-1β, IL-6 and IL-17A, at various time points following qPCR. The results suggested there were no significant difference in IL-17A expression levels in the early phase (<4 h) following infection when compared mRNA expression in 1 h and 2 h with that in 0.5 h (P_0.5 vs. 1_=0.1182 and P_0.5 vs. 2_=0.2431). However, *C. neoformans* treatment induced strong expressions of pro-inflammatory cytokines IFN-I, IFN-γ and IL-1β, which were quite different from the media mock group.

### Blockade of IFN-I signals decreases IL-17A expression levels in T cells

**Figure 2:**
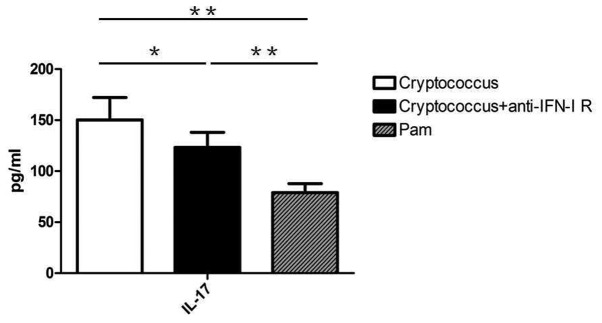
IL-17A expression levels following 7 day co-culture of mice splenocytes with *C. neoformans* in the absence or presence of anti-IFN-I-R antibodies. IL, interleukin; IFN-I-R, type-I interferon receptor.

Previous studies on IFN-I have suggested that members of the IFN-I family induce transcription of numerous target genes involved in host anti-virus and -bacterial infection (13,14). However, fungal infection is commonly recognized via the TLR2, TLR4 and TLR6 signaling pathways and does not result from the secretion of IFN-I. In the present study, a peak expression of IFN-I was noted in the early phase (<0.5 h) of *C. neoformans* infection. This transient expression of IFN-I occurred prior to the release of additional cytokines and its function was clear such as inducing synthesis of NO and activation of macrophages. To examine whether heightened IFN-I secretion was necessary for Th17 cell development and IL-17A production, the IFN-I-receptor (R) was blocked with anti-IFN-I receptor neutralizing antibodies in the co-culture model and the TLR2 agonist, Pam3CSK4, was used to activate TLR signaling pathways as the control. Notably, no significant differences in IL-17A production were observed via ELISA (data not shown). Considering that neutrophils in human PBMCs are a major source of IL-17A (13), this treatment was repeated in mice splenocytes, which have a lower number of neutrophils than PBMCs, and found that blockade of IFN-I reduced IL-17A secretion levels after 7 days of co-culture. In addition, Pam3CSK4-treated mice splenocytes did not express the high levels of IFN-I (data not shown) and IL-17A observed in the control group (Fig. 2).

### Blockade of IFN-I signals inhibits Th17 cell development in vitro

**Figure 3:**
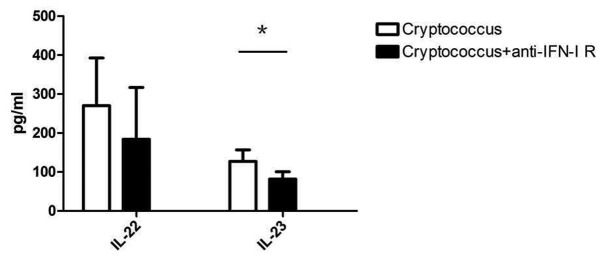
IL-22 and IL-23 expression levels following 7 day co-culture of mice splenocytes with *C. neoformans* in the absence or presence of anti-IFN-I-R antibodiess. IL, interleukin; IFN-I-R, type-I interferon receptor.

To understand IFN-I and its roles in Th17 cell development, the expression of IL-22 and IL-23 was determined in co-cultured mice splenocytes. IL-22 is the key factor in the mucosal immune response and clearance of pathogenic microbes (15). IL-23 promotes naive CD4^+^ T cell differentiation towards Th17 cells (18,19), and IL-22 and IL-23 are produced by Th17 cells. In the present model, blockade of IFN-I signals in *C. neoformans* co-cultured mice splenocytes with anti-IFN-I receptor neutralizing antibodies resulted in decreased expression levels of IL-22 and IL-23 compared with that of the control group after 7 days of culture (P=0.0453) (Fig. 3). These results suggest that the expression levels of IFN-I in the early phase of *C. neoformans* infection may have a guiding role in Th17 cell differentiation and host anti-fungal immune responses.

## Discussion

Cryptococcosis is a disease resulting from fungal infection. Although a number of cell subsets, such as neutrophils and DCs, are involved in the clearance of *Cryptococcus* spp., Th17 cells and innate cells are accepted as the major regulatory factors (16). The recognition of pathogenic microbes via PPAR family members is important to activate local inflammations in the early phase of infection. Recent studies (20–22) have shown that MyD88-mediated TLR signals are involved in the clearance of Cryptococcus via cytokine secretion; however, the mechanisms of these cytokines in immune regulation remain unclear.

In the present study, human PBMCs and heat-killed *C. neoformans* were co-cultured to mimic cryptococcal infection in human PBMCs to examine the cytokine profile at various time points. Results suggest that heat-killed *C. neoformans* increased the expression levels of IFN-I, IL-1β and IL-6 in the acute phase of infection and enhanced IL-17A production after 2 h (data not shown). Notably, the recognition of *C. neoformans* was mediated by TLR2, TLR4 and/or TLR6, which did not result in high levels of IFN-I secretion. To understand whether IFN-I is necessary in the host anti-fungal immune response, the IFN-I-R signal was blocked with anti-IFN-I-R neutralizing antibodies. This blockade of IFN-I was found to specifically reduce IL-17A expression in mice splenocytes, but had almost no effect on human PBMCs. Considering that there were numerous IL-17A-producing innate cells (such as neutrophils and γδ T cells) in human PBMCs, we speculated that IFN-I may be important for Th17 cell polarization, however, the mechanisms remain unclear. Further examination of IL-22 and IL-23 expression levels were also consistent with this hypothesis.

The common opinion regarding Th17 cell development both *in vitro* and *in vivo* suggests that the polarization of Th17 cells is regulated by IL-6 and TGF-β, which is secreted by adipocytes (23,24). However, the mechanisms may be more complex. A previous bioinformatic study of Th17 cell differentiation (25) suggested that a number of transcription factors, such as signal transducer and activator of transcription 3 (STAT3), interferon regulatory factor-4 (IRF4) and basic leucine zipper transcription factor, ATF-like, are activated much earlier than IL-17A gene transcription. In addition, a study of DC subsets identified that IRF4 transcription-factor-dependent CD103^+^ DCs specifically directed Th17 cell polarization (26). In the present study, we reported that IFN-I expression was detected in the early phase in *C. neoformans* infection but not IL-6 or IL-17 expression. In addition, the activation of IFN-I expression was required in the followed immune responses as blockade of IFN-I signal downregulated cytokines IL-17, IL-22 and IL-23 secretions *in vitro*. These finding suggest the critical roles of IFN-I in the clearance of fungi infection and provided direction for the future treatment of cryptococcosis.

## Figures and Tables

**Figure 1:**
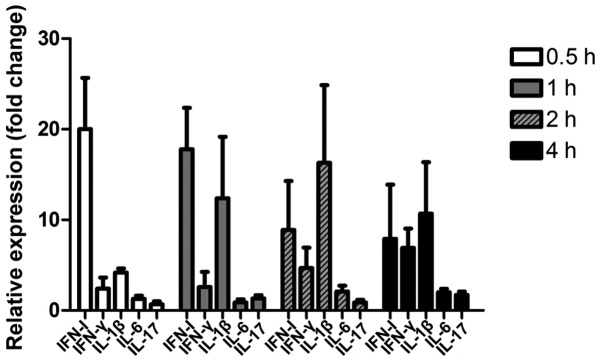
Relative expression levels of cytokines following co-culture of human peripheral blood mononuclear cells with *C. neoformans*. Data are expressed as fold change of *C. neoformans*-treated group versus media mock group. IL, interleukin.

## References

[1] Biondo C, Midiri A, Messina L, Tomasello F, Garufi G, Catania MR (2005). MyD88 and TLR2, but not TLR4, are required for host defense against *Cryptococcus neoformans*. Eur J Immunol.

[2] Nakamura K, Miyagi K, Koguchi Y, Kinjo Y, Uezu K, Kinjo T (2006). Limited contribution of Toll-like receptor 2 and 4 to the host response to a fungal infectious pathogen, *Cryptococcus neoformans*. FEMS Immunol Med Microbiol.

[3] Wormley FL, Perfect JR, Steele C, Cox GM (2007). Protection against cryptococcosis using a murine gamma interferon-producing *Cryptococcus neoformans* strain. Infect Immun.

[4] Wozniak KL, Ravi S, Macias S, Young ML, Olszewski MA, Steele C, Wormley FL (2009). Insights into the mechanisms of protective immunity against *Cryptococcus neoformans* infection using a mouse model of pulmonary cryptococcosis. PLoS One.

[5] Peck A, Mellins ED (2010). Precarious balance: Th17 cells in host defense. Infect Immun.

[6] Ley K, Smith E, Stark MA (2006). IL-17A-producing neutrophil-regulatory Tn lymphocytes. Immunol Res.

[7] Bettelli E, Carrier Y, Gao W, Korn T, Strom TB, Oukka M, Weiner HL, Kuchroo VK (2006). Reciprocal developmental pathways for the generation of pathogenic effector TH17 and regulatory T cells. Nature.

[8] Korn T, Bettelli E, Oukka M, Kuchroo VK (2009). IL-17 and Th17 cells. Annu Rev Immunol.

[9] Ferretti S, Bonneau O, Dubois GR, Jones CE, Trifilieff A (2003). IL-17, produced by lymphocytes and neutrophils, is necessary for lipopolysaccharide-induced airway neutrophilia: IL-15 as a possible trigger. J Immunol.

[10] Chang J, Burkett PR, Borges CM, Kuchroo VK, Turka LA, Chang CH (2013). MyD88 is essential to sustain mTOR activation necessary to promote T helper 17 cell proliferation by linking IL-1 and IL-23 signaling. Proc Natl Acad Sci USA.

[11] Jin B, Sun T, Yu XH, Yang YX, Yeo AE (2012). The effects of TLR activation on T-cell development and differentiation. Clin Dev Immunol.

[12] Barboza R, Câmara NO, Gomes E, Sá-Nunes A, Florsheim E, Mirotti L (2013). Endotoxin exposure during sensitization to *Blomia tropicalis* allergens shifts TH2 immunity towards a TH17-mediated airway neutrophilic inflammation: role of TLR4 and TLR2. PLoS One.

[13] Perry AK, Chen G, Zheng D, Tang H, Cheng G (2005). The host type I interferon response to viral and bacterial infections. Cell Res.

[14] Rifkin IR, Leadbetter EA, Busconi L, Viglianti G, Marshak-Rothstein A (2005). Toll-like receptors, endogenous ligands, and systemic autoimmune disease. Immunol Rev.

[15] Lilly LM, Gessner MA, Dunaway CW, Metz AE, Schwiebert L, Weaver CT, Brown GD, Steele C (2012). The β-glucan receptor dectin-1 promotes lung immunopathology during fungal allergy via IL-22. J Immunol.

[16] Wozniak KL, Hardison SE, Kolls JK, Wormley FL (2011). Role of IL-17A on resolution of pulmonary *C. neoformans* infection. PLoS One.

[17] Clifford JL, Walch E, Yang X, Xu X, Alberts DS, Clayman GL (2002). Suppression of type I interferon signaling proteins is an early event in squamous skin carcinogenesis. Clin Cancer Res.

[18] Frazer LC, Scurlock AM, Zurenski MA (2013). IL-23 induces IL-22 and IL-17 production in response to *Chlamydia muridarum* genital tract infection, but the absence of these cytokines does not influence disease pathogenesis. Am J Reprod Immunol.

[19] Yang CY, Ma X, Tsuneyama K (2013). IL-12/Th1 and IL-23/Th17 biliary microenvironment in primary biliary cirrhosis: Implications for therapy. Hepatology.

[20] Dan JM, Wang JP, Lee CK, Levitz SM (2008). Cooperative stimulation of dendritic cells by *Cryptococcus neoformans* mannoproteins and CpG oligodeoxynucleotides. PLoS One.

[21] Wang JP, Lee CK, Akalin A, Finberg RW, Levitz SM (2011). Contributions of the MyD88-dependent receptors IL-18R, IL-1R, and TLR9 to host defenses following pulmonary challenge with *Cryptococcus neoformans*. PLoS One.

[22] Redlich S, Ribes S, Schütze S, Eiffert H, Nau R (2013). Toll-like receptor stimulation increases phagocytosis of *Cryptococcus neoformans* by microglial cells. J Neuroinflammation.

[23] O’Shea JJ, Steward-Tharp SM, Laurence A, Watford WT, Wei L, Adamson AS, Fan S (2009). Signal transduction and Th17 cell differentiation. Microbes Infect.

[24] Zhou L, Littman D (2009). Transcriptional regulatory networks in Th17 cell differentiation. Curr Opin Immunol.

[25] Yosef N, Shalek AK, Gaublomme JT (2013). Dynamic regulatory network controlling TH17 cell differentiation. Nature.

[26] Persson EK, Uronen-Hansson H, Semmrich M (2013). IRF4 transcription-factor-dependent CD103(+)CD11b(+) dendritic cells drive mucosal T helper 17 cell differentiation. Immunity.

